# Should the Appendix Be Removed in All Cases Where the Abdomen Is Opened for Other Causes?

**Published:** 1908-11

**Authors:** L. C. Fischer


### SHOULD THE APPENDIX BE REMOVED IN ALL CASES WHERE THE ABDOMEN IS OPENED FOR OTHER CAUSES?

BY L. C. FISCHER, M. D.

In presenting this subject to this Association I fully realize that it is one of great importance, and one that should be thoroughly considered by the operator and by the Medical Profession. I realize that all of the organs must have some function. It is a much mooted question as to what is the function, if any, of the appendix. Different writers having different opinions. About 1847 the function of the appendix was supposed to be that of secreting fluid which kept the contents of the cecum in a fluid or semi-fluid state. From that date to the present date different functions have been attributed to it. At the present time the best authorities think that the appendix has no function, or at least no function that seems to be disturbed by its remov[illegible]

It has been my experience, that in most cases where the appendix was removed, either as the primary operation or as secondary to the opening of the abdomen that the patient has invariably been in better health. This, of course, could not be attributed entirely to the removal of the appendix where that was not the conditions will cause this improvement in health. In no case do primary operation. Of course the removal of other pathological conditions will cause this improvement in health. In no case do I know of, where removing the appendix has caused any bad effect or change in health for the worse, except in two cases where adhesions were formed, due possibly to the lack of closing over the raw surface with peritoneum, or where there was acute inflammation. On the contrary I do know of cases where the appendix was not removed at the time of opening the abdomen, where shortly afterwards it was necessary to re-open the abdomen on account of the inflammation of this appendage. Two cases that have occurred in my own work within the last four years. For the last two years it has been my rule to remove the appendix when I opened the abdomen for other causes, where the condition of the patient would allow this extra procedure. The operation is done in a very few minutes and saves the patient the possibility of opening the abdomen at another time.

The cases above referred to are as follows:

Mrs. C., age 40, upon whom I did a pan-hysterectomy, closing over all of the raw surface, except on the extreme right angle of my incision, where I had cut through the broad ligament. About three months after the first operation I was called to see her in a acute attack of appendicitis, she at that time refused surgical interference, but after the third attack allowed me to open the abdomen. Upon opening it I found the appendix very long and adherent over the entire length of the cicatrix, caused by the removal of the uterus. Upon dissecting this out and removing the appendix the patient made an uneventful recovery.

The second case, Miss C. Where I had removed the right ovary and suspended the uterus. After this operation the patient had a rising temperature for a few days and some adhesions were formed around the stump. Nine months later I was called to see this patient with an acute attack of appendicitis, operated within the first 12 hours, found the appendix with its distal end adherent by adhesions to the stump where the ovary had been removed.

These two cases and their severity fully demonstrate the dangers of adhesions of the appendix where work has been done in the pelvis.

During the last 12 months it has been my privilege to see Dr. H. C. Coe, of New York do quite an amount of surgery and I do not remember in a single case where he did not remove the appendix, where the condition of the patient would allow. Dr. John B. Deaver, of Philadelphia after completing his primary operation looks up at his audience and says in a matter of fact tone, "Now, gentlemen, to complete this operation I will remove the appendix."

In order that I might present this subject to you from both sides I have written to some of the most noted surgeons of this country for their rules, the following questions were asked:

(1) Do you remove the appendix if you open the abdomen for any other cause?

(2) Have you had to re-open the abdomen for appendectomy upon patients who you had operated for other causes, if so did you find any adhesions around the appendix that you thought could be due to previous operations?

(3) Do you know of any change in health in patients in whom the appendix has been removed, where the appendix was not the primary cause of the operation?

To these I have received the following replies: Dr. E. C. Davis, Atlanta, Ga.

(1) Yes, if the patients' consent is gained and her condition justifies the additional operation.

(2) Yes, in a number of instances with marked adhesions around the appendix.

(3) Yes, I believe in a number of instances the general health of the patient has been improved, and I recall no instance any ill effect from the removal of the appendix."

Dr. Joe Pierce, of Philadelphia:

"(1) Yes.

(2) Yes.

(3) Yes, improved. The appendix is a faulty piece of anatomy, always charged with the intestinal flóra and never healthy. Ten or more per cent. of pelvic suppurations are complicated by the appendix and head of the cecum. It is often overlooked post operation complications and some deaths, or reopening necessary."

Dr. Geo. H. Noble, Atlanta, Ga.

"(1) I do if the appendix is diseased or mutilated, otherwise do not remove it.

(2) Have operated on cases done by other surgeons, histories unsatisfactory, adhesions variable.

(3) No. Removal of healthy appendix does not seem to cure any thing."

Dr. John A. Wyeth, New York:

"(1) Never, unless diseased.

(2) Never.

(3) No. The proportion of diseased appendices is so small thtat its removal in the course of an operation for other cause than appendicitis is, in my opinion, scarcely worthy of consideration. If the operation were in the immediate neighborhood of this organ, and its peculiar structure and location should convince the operator that it was more than ordinarily susceptible to disease, it might justify its removal. However, no severe operation should be prolonged by the removal of an appendix not diseased."

Dr. Howard A. Kelly, Baltimore:

"(1) Always, if any suspicions of disease or if the patient requests it.

(2) In several instances; see my work on diseases of the Vermiform Appendix. Look for case under Hunter McGuire's name, etc.

(3) Have never known any bad effects from removing the appendix; in many instances an unsuspected disease of the appendix has been a source of trouble. In conclusion, I do not believe in removing the appendix in every case, but I do believe in widely extending the limits of this simple operation."

Dr. Howard J. Williams, Macon, Ga:

"(1) If there is any indication of removal of the appendix, during other abdomnal operation—I do not hesitate in doing so; in every abdominal operation, before closing the abdomin I examine the appendix, finding adhesions or other conditions which would make it advisable to remove it, I do so before closing the abdominal wound.

(2) I cannot recall such a condition.

(3) No; if the patient were not told that the appendix had been removed, I doubt that he or she could ever discover any change or symptoms."

Dr. W. T. Bull, New York:

"(1) Uusually in hysterectomy or operations of right broad ligaments.

(2) No. Have had several cases of acute appendicitis following hysterectomy or other operations where adhesions of appendix seemed to be the cause of the inflammation.

(3) No."

Dr. John B. Murphy, Chicago:

"(1) I do not remove the appendix in every abdominal operation, only when the appendix shows evidence of existing disease or that it has been previously inflamed. I examine the appendix in every laparotomy.

(2) Once only, one week after the extirpation of a fibroid. It was acutely infected and gangrenous.

(3) I know of no case. Do not attribute improvement of patients to remove a appendix where appendix was not found to be actually diseased."

Dr. H. C. Coe, New York:

"(1) Yes.

(2) Yes.

(3) Intestinal fistula in one case."

Dr. Robt. T. Morris, New York:

"(1) Leave the appendix alone until it is infected, and then lose no time in having it inspected.

(2) I can recall two or three cases in which adhesions following previous pelvic work seemed to have been influential in the development of appendicitis later.

(3) No unfavorable change in health in my own patients, so far as I know, but on the contrary most satisfactory changes for the better. It may be that patients with unfavorable results do not return to me for report. I make this comment for the reason that very many patients operated upon by others come to me for repair of hernas and separation of adhesions. The mere fact of absence of the appendix has no bearing because it is abscess in most elderly people, and in patients who have had distinctive inflammation of the appendix."

You will see from the best authorities that a great many of them do remove the appendix and especially so if it shows the least evidence of inflammation. Dr. Robt. T. Morris calls attention to the fact that the appendix is atrophied in old people and also to the obsence of the appendix in distinctive inflammation. Several times on opening the abdomen have I found what Dr. John B. Deaver chooses to term "Suppurative amputation." This condition is the result of previous inflammation and results in a complete occlusion of the lumen of the appendix with subsequent atrophy. In these cases the patient gives a history of repeated previous attacks and invariably agrees to have the appendix removed for fear of at some future time they may have another attack. I believe the time is not far distant when the majority of the operators will consider it their duty to in all cases remove the appendix, where the conditions of the patient will allow and where there is no suppuretive inflammation.

I have watched the patients upon whom I have operated for the last two years, where it was possible for me to do so, and no single instance have I found that they have suffered any ill effect from the removal of the appendix, to the contrary I believe they have been materially benefitted.

